# An Italian Delphi study to evaluate consensus on adjuvant endocrine therapy in premenopausal patients with breast cancer: the ERA project

**DOI:** 10.1186/s12885-018-4843-2

**Published:** 2018-09-27

**Authors:** Giacomo Pelizzari, Grazia Arpino, Laura Biganzoli, Saverio Cinieri, Michelino De Laurentiis, Lucia Del Mastro, Angelo Di Leo, Stefania Gori, Valentina Guarneri, Paolo Marchetti, Fabio Puglisi

**Affiliations:** 1 Department of Clinical Oncology, IRCCS CRO Aviano - National Cancer Institute, Aviano, Italy; 2 Department of Medicine (DAME), University of Udine, Udine, Italy; 3 Department of Clinical Medicine and Surgery, University Federico II, Naples, Italy; 4 Sandro Pitigliani Medical Oncology Department, Hospital of Prato, Istituto Toscano Tumori, Prato, Italy; 5 Medical Oncology Division & Breast Unit, Senatore Antonio Perrino Hospital, Brindisi, Italy; 6 Breast Oncology Department, Istituto Nazionale Tumori “Fondazione G. Pascale”, Napoli, Italy; 7 Department of Internal Medicine, University of Genova, Ospedale Policlinico San Martino, Genoa, Italy; 8 Medical Oncology Unit, IRCCS Ospedale Sacro Cuore Don Calabria, Negrar, Italy; 9 Division of Medical Oncology 2, Instituto Oncologico Veneto IRCCS, Padova, Italy; 10 Department of Surgery, Oncology and Gastroenterology, University of Padova, Padova, Italy; 11 Medical Oncology, Sapienza, University of Rome, and IDI-IRCCS, Rome, Italy

**Keywords:** Breast cancer, Premenopausal patients, Adjuvant endocrine therapy, Ovarian function suppression, LHRHa, Tamoxifen, Aromatase inhibitor

## Abstract

**Background:**

Several trials evaluated the role of ovarian function suppression for the adjuvant treatment of premenopausal patients with hormone receptor-positive early breast cancer. Based on the results of the SOFT and TEXT trials, international guidelines recommend the addition of ovarian function suppression to standard adjuvant endocrine therapy for patients at higher risk of relapse.

**Methods:**

The ERA project (Evaluation of Risk factors in the Adjuvant treatment of breast cancer in premenopausal patients) was devised with the objective of obtaining a consensus on the identification of risk factors and the use of ovarian function suppression in the adjuvant treatment of these women. To this aim, a panel of 31 Italian oncologists with expertise in breast cancer participated in a Delphi consensus study in June 2017.

**Results:**

A total of 29 statements related to prognostic factors, therapeutic strategies and ovarian function suppression were defined and voted to gain final consensus. For each topic we report data supporting the acquired consensus and the relevant issues discussed.

**Conclusions:**

The SOFT and TEXT trials have changed the standard adjuvant treatment of premenopausal patients with hormone receptor-positive early breast cancer, but the available treatment options require a careful risk assessment and toxicities evaluation to ensure the greatest clinical benefit for each patient.

## Background

Breast cancer represents the most frequently diagnosed cancer in premenopausal women, with about 20% of all breast cancers diagnosed before age 50 [1]. Most of these tumors are hormone receptors-positive (HR-positive), so that adjuvant hormonal therapy is usually recommend to reduce the risk of recurrence after radical surgery [2, 3]. For decades, tamoxifen for 5–10 years has represented the standard adjuvant endocrine therapy for premenopausal women with HR-positive early breast cancer (EBC), with a significant reduction in breast cancer mortality for up to 15 years from diagnosis [4]. Recently, the SOFT and TEXT trials have revaluated the role of ovarian function suppression (OFS) in the treatment of these women, combining luteinizing hormone-releasing hormone analogs (LHRHa) with tamoxifen or aromatase inhibitors (AIs), and providing new data for the management of premenopausal patients with EBC [5–7]. On the basis of these trials, international guidelines recommend the use of OFS as a component of standard endocrine therapy for premenopausal women at high risk of relapse, pointing out the critical role of risk assessment for these patients [8–10]. Therefore, one of the major challenges in choosing the optimal adjuvant treatment of premenopausal HR-positive EBC patients is the identification of reliable prognostic factors and their contribution in recurrence risk. Indeed, the absolute benefit of adjuvant treatments depends on the estimated risk of cancer recurrence [11], thus generating debate in defining a treatment algorithm for individual patients since clinical trials mainly focus on treatment efficacy in the general population.

The ERA project (Evaluation of Risk factors in the Adjuvant treatment of breast cancer in premenopausal patients) implemented a Delphi model to gain consensus on the evaluation of risk factors and the use of OFS in the adjuvant treatment of premenopausal patients with ER-positive EBC.

## Methods

The Delphi consensus model applied to this study consisted of three key phases. During the first phase, a Steering Committee including 10 experts in breast cancer (BC) was asked to define relevant statements on the topic at hand. After two advisory boards, the Steering Committee defined 29 preliminary recommendation statements on prognostic factors, adjuvant endocrine treatment and LHRHa therapy in premenopausal patients with ER-positive EBC, based on available published data. In the second phase, a web-based survey was distributed to a panel of 40 oncologists with expertise in BC. Overall, 31 recipients completed the online consensus survey (hereinafter referred to as the expert panel). The panellists were asked to express anonymously their level of agreement with each statement, using a five point Likert scale (where 1 = completely disagree; 2 = slightly disagree; 3 = partially agree; 4 = agree; 5 = completely agree) [12]. A consensus was deemed as achieved if either the sum of answers 1 and 2 (negative), or 3, 4 and 5 (positive) exceeded 66%, as described in previous studies conducted with this method [13, 14]. The third phase was held during a consensus conference in Rome on 19 June 2017. The expert panel (31 members) and the Steering Committee (10 members) were invited to discuss in public sessions the results of the online consensus survey and voted again for those statements with partial consensus (mainly level 3 agreement) or negative consensus. Of the 41 members, 21 took part in the final meeting.

## Results

During the consensus conference, 27 statements met a positive consensus, whereas 2 statements did not reach the threshold for positive agreement. An additional statement was defined during the third phase and gained positive consensus after the vote of the panellists. Overall, 28 statements reached a positive consensus (Table 1) and were clustered in five conclusive highlights (1–5) related to 3 main topics: prognostic groups, therapeutic strategy and ovarian function suppression. For each topic we report data supporting the acquired consensus and relevant issues discussed by the panellists, including reasons for negative consensus if occurred.

**Table 1:** Delphi model results

Topic	Statement	Level of agreement/disagreement					Positive Consensus^a^
		Completely disagree	Slightly disagree	Partially agree	Agree	Completely agree	
Prognostic groups	1. The risk of recurrence in premenopausal women with HR-positive EBC depends on prognostic factors.						
	The following prognostic factors should be considered for risk assessment:						
	A. Tumor stage (T and N)	0%	0%	10%	32%	58%	100%
	B. Tumor grade	0%	0%	16%	39%	45%	100%
	C. HER2 overexpression	0%	0%	0%	23%	77%	100%
	D. Age	3%	3%	39%	32%	23%	94%
	E. Gene expression profile	0%	3%	39%	42%	16%	97%
	F. Ki-67	0%	0%	10%	39%	51%	100%
	G. ER and PgR expression levels	0%	3%	7%	35%	55%	97%
	H. Histology (ductal vs. lobular)	38%	52%	5%	0%	5%	10%
	2. Predictive and prognostic factors guide the choice of adjuvant endocrine treatment in premenopausal women with HR-positive EBC.						
	The following factors guide the choice of treatment in premenopausal patients who are candidates for adjuvant endocrine therapy:						
	A. Tumor stage (T and N)	0%	6%	20%	29%	45%	94%
	B. Tumor grade	3%	7%	16%	52%	22%	90%
	C. HER2 overexpression	0%	10%	12%	39%	39%	90%
	D. Age	0%	10%	19%	39%	32%	90%
	E. Gene expression profile	0%	13%	32%	42%	13%	87%
	F. Ki-67	0%	3%	23%	39%	35%	97%
	G. ER and PgR expression levels	0%	3%	6%	39%	52%	97%
	H. Histology (ductal vs. lobular)	33%	52%	5%	0%	10%	15%
Therapeutic strategy	3. For premenopausal women with HR-positive EBC at low risk of recurrence, adjuvant tamoxifen is the recommended adjuvant treatment.						
	A. The addition of OFS to tamoxifen or exemestane did not show clinical benefit in terms of DFS, DDFS, BCFI and OS in low–risk patients.	6%	10%	13%	45%	26%	84%
	B. The addition of OFS to standard endocrine therapy could be associated with worse adverse effects, according to patients’ age.	3%	10%	32%	36%	19%	87%
	C. If tamoxifen is contraindicated, OFS with or without exemestane should be considered.	0%	0%	6%	42%	52%	100%
	4. The addition of OFS to standard adjuvant endocrine therapy is recommended for premenopausal women with HR–positive EBC at intermediate or high risk of recurrence.						
	A. The addition of OFS to tamoxifen reduces the risk of breast cancer recurrence.	0%	3%	13%	52%	32%	97%
	B. OFS plus exemestane reduced the risk of breast cancer recurrence compared to OFS plus tamoxifen.	0%	0%	23%	45%	32%	100%
	C. OFS plus exemestane reduced the risk of breast cancer recurrence compared to tamoxifen alone.	0%	3%	6%	52%	39%	97%
	D. The combination of OFS and tamoxifen may be an alternative to OFS plus AIs for patients at intermediate risk of breast cancer recurrence.	0%	3%	45%	32%	20%	97%
Ovarian function suppression	5. The standard duration of adjuvant OFS for premenopausal women with HR-positive EBC is 5 years, with LHRHa therapy administered on a monthly basis.						
	A. Available data on OFS come from clinical trials in which LHRHa therapy was administered on a monthly basis.	0%	0%	6%	55%	39%	100%
	B. Adjuvant endocrine treatment with LHRHa therapy plus AIs may result in incomplete ovarian suppression.	0%	26%	16%	32%	26%	74%
	C. Potential predictive factors for suboptimal ovarian suppression are high BMI, no prior chemotherapy, low baseline FSH and LH levels.	0%	13%	23%	45%	19%	87%
	D. Surgical oophorectomy could be an alternative to LHRHa therapy, according to patients’ preferences.	3%	7%	16%	39%	35%	90%
	E. LHRHa therapy should start preferably on the second day of the menstrual cycle.	0%	19%	13%	42%	26%	81%
	F. AIs should be administered at least 4 weeks after the first dose of LHRHa therapy, when associated with OFS.	0%	4%	32%	32%	32%	96%
	G. Perimenopausal patients with chemotherapy-induced amenorrhea should receive OFS if baseline FSH and E2 levels are not in the postmenopausal range.	6%	6%	0%	28%	60%	88%

Abbreviations: *HR* hormone receptors, *EBC* early breast cancer, *HER2* Human epidermal growth factor receptor 2, *ER* estrogen receptor, *PgR* progesterone receptor, *OFS*, ovarian function suppression, *DFS* disease-free survival, *DDFS* distant disease-free survival, *BCFI* breast cancer-free interval, *OS* overall survival, *AIs* aromatase inhibitors, *LHRHa* luteinizing hormone-releasing hormone analogs, *BMI* body mass index, *FSH* follicle-stimulating hormone, *LH* luteinizing hormone, *E2* beta-2-estradiol

^a^A consensus was deemed as achieved if either the sum of answers 1 and 2 (negative), or 3, 4 and 5 (positive) exceeded 66%

### Prognostic groups

#### The risk of recurrence in premenopausal women with HR-positive EBC depends on prognostic factors

A number of tumor- and patient-related characteristics have been found relevant in predicting the risk of recurrence or death from EBC. Eight prognostic factors were reviewed and individually voted by the panellists. High level of agreement was observed during the discussion, with seven out of eight statements meeting a positive consensus with more than 94% positive agreement according to the pre-specified criteria (Table 1). As discussed by the panellists, traditional prognostic factors include axillary nodes involvement [15] and tumor size [16, 17]. Tumor grade [18–20], and proliferative markers (e.g. Ki-67 expression assessed by immunostaining) [21, 22] provide additional prognostic information and should be routinely considered for risk assessment. However, the lack of a reliable cut-off point for Ki-67 evaluation and its low inter-laboratory reproducibility were considered relevant issues by the panellists, suggesting as a better reference the median value of the local laboratory [23–25]. Additional biological features, including the expression levels of estrogen-receptor (ER), progesterone-receptor (PgR) and the overexpression of the human epidermal growth factor receptor 2 (HER2) are both prognostic and predictive in EBC [26–32]. On this latter topic, the prognostic relevance of HER2 overexpression, biologically associated with increased tumor aggressiveness and higher rates of recurrence, has radically changed with the use of adjuvant anti-HER2 targeted therapies, insuring improved outcomes even when compared with luminal lower-stage tumors [33, 34]. Taking into account the ER/PgR expression levels, recent data suggest that BC with ER expression inferior to 10% are more similar to triple-negative breast cancer (TNBC) than luminal disease, with worse survival outcomes and limited advantage from adjuvant endocrine therapy [35]. Additionally, ER-positive/PgR-negative tumors seem to present worse outcomes compared to ER/PgR-positive EBC [36]. This data suggest that low levels of expression of both ER and PgR may indicate a more aggressive biology and a lower likelihood of benefit from adjuvant endocrine treatment. Nevertheless, a 1% cut-off is still recommended for estrogen receptor positivity by the American Society of Clinical Oncology (ASCO)/College of American Pathologists (CAP) guidelines [37].

Histological assessments are often used as surrogate to define BC molecular subtypes in clinical practice. In this regard, multigene assays such as Oncotype DX™ and MammaPrint® (a 21-gene recurrence score and a 70-gene signature, respectively) are considered to add prognostic information, particularly for avoiding adjuvant chemotherapy at low risk women, when proliferative markers do not provide conclusive clues even if matched with classic clinico-pathological factors [38–40]. Of note, the eighth edition of the primary Tumor, lymph Node, and Metastasis (TNM) classification of the American Joint Commission on Cancer (AJCC) incorporated biologic prognostic and predictive factors (tumor grade, HR and HER2 expression, multigene assays) into EBC staging [41]. In this respect, the panel endorsed the use of the eighth AJCC classification in clinical practice starting from January 2018, since it offers more accurate prognostic information. Prospective validation data for EBC patients were published after this consensus study and confirmed 29.5% of patients upstaged and 28.1% downstaged with the new AJCC classification [42].

Considering patient-related factors, younger age has been associated with worse outcome in HR-positive EBC [43], probably due to suboptimal estrogen inhibition, less therapeutic adherence, and differences in tumor biology. However, as widely discussed by the panellists, younger patients should not be considered high-risk per se, but age should be evaluated with other prognostic factors during risk assessment.

A negative consensus was reached when considering the prognostic relevance of tumor histology (ductal vs. lobular) in HR-positive EBC patients (38% completely disagree, 52% slightly disagree). As argued by the panellists, lobular breast cancer typically presents with good prognostic features (G1–2, low Ki-67, HR-positive and usually HER2-negative) [44], but no clear prognostic advantage has been ever shown, with some contrasting evidence on long-term outcomes [44, 45].

In conclusion, the panellists established that the following prognostic factors should be considered for risk assessment:

A. Tumor stage (T and N)
B. Tumor grade
C. HER2 overexpression
D. Age
E. Gene expression profile
F. Ki-67
G. ER and PgR expression levels

The seven above-mentioned prognostic factors were considered as independent statements and separately voted. An additional element, histology (ductal vs. lobular), did not reach the positive consensus and was excluded from the list (Table 1).

#### Predictive and prognostic factors guide the choice of adjuvant endocrine treatment in premenopausal women with HR-positive EBC

After the identification of significant prognostic factors in premenopausal women with HR-positive EBC, panel members were asked about the clinical relevance of these elements in guiding the therapeutic choices. There are different aspects to consider when assessing this issue, including a missing agreement on the threshold for recommending adjuvant chemotherapy, and a variable magnitude of benefit deriving from different endocrine therapies. The statements discussed below focused on which prognostic factors should be considered for adjuvant treatment choice, stratifying patients for high-risk and low-risk features, without defining a specific clinical setting of applicability. In this respect, the panellists did not define any particular combination of these factors to give indications about low or high-risk definition, since specific clinical considerations need to be discussed on a case-by-case basis. Particular clinical scenarios will be reviewed further on. All the prognostic items selected above were confirmed relevant for treatment choice, with a slightly inferior but still solid overall agreement among the panellists (more than 87% positive agreement according to the pre-specified criteria; see Table 1).

During the public discussion, the panel addressed some open questions on factors predicting adjuvant endocrine treatment benefit. Firstly, as previously discussed, international guidelines are not concordant on a common threshold for HR positivity (ER/PgR expression > 1% vs. > 10%) [37, 38]. Secondly, considering HER2-positive luminal BC, the panellists warned about possible crosstalk between the estrogen receptor and HER2 pathways, with conflicting evidences referring on a potential detrimental effect of tamoxifen in triple-positive BC [46–48]. Prospective data are too limited to draw final considerations, although some panellists prudentially assumed that the use of tamoxifen as the sole adjuvant therapy might not be appropriate for premenopausal patients with HER2-positive luminal BC. Finally, the panel commented on the positive consensus reached over the use of genomic assays to tailor adjuvant treatments for premenopausal patients (13% slightly disagree, 32% partially agree, 55% agree/completely agree). Clinical validation of these assays is becoming reliable, supporting their use in clinical practice, particularly to avoid chemotherapy for patients with node-negative luminal EBC [39, 40]. The elevated prevalence of partial agreement with this statement was mainly explained by the limited use of these assays in some Italian centres, and negative or weak recommendations for their use for very low–risk or node–positive patients with luminal EBC [38, 49].

The panellist concluded that the following factors should guide the choice of treatment in premenopausal patients who are candidates for adjuvant endocrine therapy:

A. Tumor stage (T and N)
B. Tumor grade
C. HER2 overexpression
D. Age
E. Gene expression profile
F. Ki-67
G. ER and PgR expression levels

The seven above-mentioned factors were considered as independent statements and separately voted (Table 1). An eighth element, histology (ductal vs. lobular), did not reach the positive consensus and was excluded from the list (33% completely disagree, 52% slightly disagree).

### Therapeutic strategy

#### For premenopausal women with HR-positive EBC at low risk of recurrence, adjuvant tamoxifen is the recommended adjuvant treatment

The first clinical issue addressed by the panellists was to define the best adjuvant endocrine therapy for low-risk premenopausal patients with HR-positive EBC. To gain consensus on the best therapeutic strategy for these women, three statements were discussed and voted (A-C, see Table 1). Each statement will be separately reviewed for a better comprehension of the panel debate on the available evidences:

A. *The addition of OFS to tamoxifen or exemestane did not show clinical benefit in terms of disease-free survival (DFS), distant disease-free survival (DDFS), breast cancer-free interval (BCFI) and overall survival (OS) in low-risk patients*

Several studies have tried to provide evidences for the use of OFS alone or in addition to standard adjuvant therapies, including chemotherapy and tamoxifen, without drawing definitive results [50–52]. Two randomized phase III trials have further examined the combination of OFS with standard adjuvant tamoxifen. The E-3193 INT-0142 trial enrolled 345 low-risk premenopausal women with HR-positive EBC (83% pT1, pN0, median age 45 years, no adjuvant chemotherapy allowed) and no benefit was shown with the addition of OFS to tamoxifen (DFS HR: 1.17; 95% CI, 0.64–2.12; *p* = .62; OS HR: 1.19; 95% CI 0.52–2.70; *p* = .67) [53]. Similarly, the SOFT trial randomized 3066 premenopausal patients to receive 5 years of adjuvant tamoxifen, OFS plus tamoxifen, or OFS plus exemestane. The primary analysis aimed at comparing the benefit of OFS plus tamoxifen with adjuvant tamoxifen alone. With a median follow-up of 5.6 years, no advantage was observed on DFS (HR: 0.83; 95% CI 0.66–1.04; *p* = .10), DDFS (HR: 0.88; 95% CI, 0.66–1.18; *p* = .40), BCFI (HR: 0.81; 95% CI, 0.63–1.03; *p* = .09), and OS (HR: 0.74; 95% CI, 0.51–1.09; *p* = .13), especially for low-risk patients (no adjuvant chemotherapy, tumor ≤2 cm, axillary node negative), who showed a 5-year DFS rate higher than 95% with adjuvant tamoxifen alone [5]. Therefore, the panel concluded that tamoxifen should be still considered the standard of care for HR-positive EBC with a low risk of recurrence according to SOFT data. An update analysis after a median follow-up of 8 years was presented subsequently this consensus study, showing a significant benefit on DFS (HR: 0.76; 95% CI, 0.62–0.93; *p* = .009), BCFI (HR: 0.76; 95% CI, 0.61–0.95; *p* = 0.01) and OS (HR: 0.67; 95% CI, 0.48–0.92; *p* = .01) but not on DDFS (HR: 0.86; 95% CI, 0.66–1.13; *p* = .28) with the addition of OFS to tamoxifen in the entire trial population. Although no influence on relative treatment effects was seen according to chemotherapy prescription, more than 97% of patients in SOFT who did not receive chemotherapy were still free of distant recurrent and alive at 8 years regardless the treatment group. Furthermore, in subgroup analyses a non-significant difference on DFS (HR: 0.76; 95% CI, 0.52–1.12), DDFS (HR: 1.09; 95% CI, 0.46–2.57), BCFI (HR: 0.83; 95% CI, 0.52–1.32), and OS (HR: 1.96; 95% CI, 0.67–5.73) was detected in this low-risk cohort for OFS plus tamoxifen rather than tamoxifen alone [7]. In conclusion, according to these data, tamoxifen alone may still represent a reasonable adjuvant therapeutic option for patients with low-risk clinico-pathological features and HR-positive EBC.

B. *The addition of OFS to standardendocrine therapy could be associated with worse adverse effects, according to patients’ age*

In the SOFT trial, an increment of severe or life-threatening and disabling adverse events (31.3% vs. 23.7%) was observed with the addition of OFS to tamoxifen. Patients experienced worse endocrine-related symptoms such as hot flushes, musculoskeletal disorders, sweating, sleep disturbance, vaginal dryness and loss of sexual interest, particularly during the first 2 years of treatment [5, 54]. Moreover, according to TEXT data, sexual dysfunctions (vaginal dryness, decreased libido, dyspareunia) were more frequent when adding OFS to exemestane rather than tamoxifen, together with a higher incidence of musculoskeletal symptoms among which osteoporosis (13.2% vs. 6.4%) and fractures (6.8% vs. 5.2%) [6]. Lastly, the addition of OFS did not seem to affect global cognitive function after 1 year of treatment [55]. The risk of bone loss and osteoporosis represents a relevant concern for premenopausal women candidate to OFS, thus every patient should receive at least a baseline bone assessment and vitamin-D supplementation if needed. Therefore, the decision on the optimal adjuvant endocrine therapy should always consider treatment toxicities, which must be discussed with patients according to their preferences and comorbidities, even during the adjuvant treatment.

C. *If tamoxifen is contraindicated, OFS with or without exemestane should be considered*

For patients candidate for adjuvant endocrine therapy who are not eligible to tamoxifen (e.g. for contraindications or severe side effects), a reasonable option is the use of OFS with or without exemestane according to the risk of recurrence.

#### The addition of OFS to standard adjuvant endocrine therapy is recommended for premenopausal women with HR-positive EBC at intermediate or high risk of recurrence

The panellists were asked to focus on the use of OFS in patients at intermediate or high risk of recurrence. To untangle this topic, they reviewed the SOFT and TEXT data and obtained positive consensus over the following four statements (A-D; see Table 1). High level of agreement was observed during the discussion, with all the statements meeting a positive consensus with more than 97% positive agreement according to the pre-specified criteria (Table 1):

A. *The addition of OFS to tamoxifen reduces the risk of breast cancer recurrence*

Considering the pre-planned subgroup analysis of the SOFT trial, with a median follow-up of 5.6 years, improved outcomes were observed with the addition of OFS to tamoxifen among high-risk patients who received adjuvant chemotherapy and remained premenopausal at the end of treatment (3.6% absolute improvement in 5-year DFS rate, HR: 0.82, 95% CI, 0.64–1.07, *p* = .96; 3.6% absolute improvement in 5-year OS rate, HR: 0.64; 95% CI, 0.42–0.96; *p* = .03) and in HER2 positive tumors (12.4% absolute improvement in 5-year DFS rate, HR 0.42; 95% CI, 0.22–0.80; *p* = .04). After correction for prognostic factors, the combination of OFS plus tamoxifen was associated with a reduced hazard of recurrence, second invasive cancer, or death as compared to tamoxifen alone (HR: 0.78; 95% CI, 0.62–0.98; p = .03) [5]. Taking into consideration these results, the panellists agreed with the statement that the addition of ovarian suppression to tamoxifen is associated with reduced risk of breast cancer recurrence in patients at intermediate or high risk of recurrence (e.g. patients warranting adjuvant chemotherapy who remain premenopausal afterward). After the conclusion of this Delphi consensus study, an update of the SOFT trial with extended follow-up was presented. A 4.2% absolute gain in 8-year DFS rate (83.2% vs. 78.9%; HR: 0.76; 95% CI, 0.62–0.93, *p* = .009) and a 1.8% absolute gain in 8-year OS rate (93.3% vs 91.5%; HR: 0.67; 95% CI, 0.48–0.92; *p* = .01) were seen among patients receiving OFS plus tamoxifen rather than tamoxifen alone. The benefit of OFS addition was even higher for those patients who had received adjuvant chemotherapy (5.3% absolute improvement in 8-year DFS rate, HR: 0.76, 95% CI, 0.60–0.97; 4.3% absolute improvement in 8-year OS rate, HR: 0.59; 95% CI, 0.42–0.84), confirming the recommendation expressed by the consensus panel [7].

This is also concordant with a recent meta-analysis including data from about 6000 premenopausal patients treated with tamoxifen with or without OFS, in which the addition of OFS after adjuvant chemotherapy significantly improved OS, with a 24% mortality reduction (*p* = .03) [52].

B. *OFS plus exemestane reduced the risk of breast cancer recurrence compared to OFS plus tamoxifen*

The joint analysis of the SOFT and TEXT trials collected data from 4690 patients coming from both the studies to compare the use of OFS in association with tamoxifen or exemestane in premenopausal women with HR-positive EBC. After a median follow up of 68 months, a 3.8% gain in terms of 5-year DFS rate was observed among patients randomized to OFS plus exemestane rather than OFS plus tamoxifen (91.1% vs. 87.3%, HR 0.72, 95% CI, 0.60–0.85, *p* < .001), with a significantly higher 5-year BCFI rate (92.8% vs. 88.8%, HR 0.66, 95% CI, 0.55–0.80, *p* < .001) and 5-year DDFS rate (93.8% vs. 92%, HR 0.78, 95% CI, 0.62–0.97 *p* = .02), but no difference in OS was observed [6]. Interestingly, the authors were able to quantify the absolute treatment effect in both SOFT and TEXT trials with the adoption of a composite risk score based on prognostic clinico-pathological characteristics: an improvement ranging from 10 to 15% of the 5-year BCFI rate was observed with the combination of exemestane plus OFS compared with tamoxifen plus OFS or tamoxifen alone for patients at high risk of recurrence (age < 35, grade 2–3 tumors, high Ki-67, > 4 nodes involved, adjuvant chemotherapy), but a lower or minimal benefit was seen for patients at intermediate or minimal risk (5% and < 3%, respectively) [11].

These data were confirmed by the updated analysis of the SOFT and TEXT trials performed after 9 years of median follow-up, published after the consensus acquired in this study. The use of OFS plus exemestane vs. OFS plus tamoxifen was associated with a 4% absolute improvement in 8-year DFS rate (HR 0.77; CI 95%, 0.67–0.90; p < .001), a 2.1% absolute improvement in 8-year DDFS rate (HR 0.80; CI 95%, 0.66–0.96; p = .02) and a 0.1% non-significant gain in 8-year OS rate (HR 0.98; CI 95%, 0.79–1.22; *p* = .84) [7].

C. *OFS plus exemestane reduced the risk of breast cancer recurrence compared to tamoxifen alone*

The SOFT trial showed a significant benefit in terms of 5-year DFS rate for high-risk patients treated with tamoxifen plus OFS rather than tamoxifen alone, with a further improvement in the group who received exemestane plus OFS [5]. Of note, in the SOFT trial the comparison between OFS plus exemestane and tamoxifen alone became a secondary endpoint after the protocol amendment allowing the joint analysis with the TEXT trial, but the panellists still considered these data sufficient to establish a superiority of OFS plus exemestane to tamoxifen alone. Furthermore, subsequently to this consensus, the updated analysis of the SOFT trial after 8 years of median follow-up confirmed a significant benefit on DFS (HR 0.65; CI 95%, 0.53–0.81), DDFS (HR 0.73, 95% CI, 0.55–0.96) but not on OS (HR 0.85; CI 95%, 0.62–1.15) for OFS plus exemestane vs. tamoxifen alone in the whole trial population [7].

D. *The combination of OFS and tamoxifen may be an alternative to OFS plus AIs for patients at intermediate risk of breast cancer recurrence*

For premenopausal patients with intermediate risk of recurrence the panel estimates the benefit of exemestane plus OFS over tamoxifen plus OFS to be only moderate, even if it is difficult to classify intermediate-risk patients. According to the composite risk score by Regan et al. the BCFI benefit in this cohort is approximately 5% at 5 years [11]. Therefore, toxicities trade-off should be carefully discussed while considering the available therapeutic options, and OFS plus tamoxifen might be the preferred combination for some women. Nevertheless, some panellists believe that the intensity of adverse events associated with these treatments are mainly related to OFS itself, regardless the companion endocrine therapy, thus recommending the proven most effective combination (OFS plus exemestane), and not the one supposed to be slightly better tolerated (OFS plus tamoxifen) also for women at intermediate risk of recurrence.

### Ovarian function suppression

#### The standard duration of adjuvant OFS for premenopausal women with HR-positive EBC is 5 years, with LHRHa therapy administered on a monthly basis

In this final section the panellists were asked to consider OFS use in clinical practice, taking into account some open questions on the routinely use of LHRHa therapies. Most of the suggestions made by the panel were directly dictated from SOFT and TEXT trials recommendations on LHRHa administrations and treatment duration, while others just mirror the best clinical practice. The following statements were voted by the panellists and gained positive consensus (Table 1):

A. *Available data on OFS come from clinical trials in which LHRHa therapy was administered on a monthly basis*

In both SOFT and TEXT trials ovarian suppression was achieved by using triptorelin at a dose of 3.75 mg administered by intramuscular injection every 28 days for 5 years [5, 6]. Alternative ovarian ablation strategies allowed in both studies were surgical oophorectomy and ovarian radiation therapy. The optimal duration of LHRHa therapy should also take into account side effects, patient preferences and pregnancy plans and some panellists believe that the duration of OFS should be carefully evaluated for patients older than 50 years approaching natural menopause. The efficacy and safety of a 3-monthly administration of LHRHa have not been prospectively investigated and should not be recommended.

B. *Adjuvant endocrine treatment with LHRHa therapy plus AIs may result in incomplete ovarian suppression*

A prospective sub-study of the SOFT trial (SOFT-EST) aimed at evaluating estrogen levels in 112 patients receiving LHRHa plus tamoxifen or exemestane. Overall, 34.2% of patients receiving exemestane plus triptorelin had at least one post-baseline beta-2-estradiol (E2) level considered inconsistent with postmenopausal status [56]. Even considering these results, the panellists did not recommend regular monitoring of follicle-stimulating hormone (FSH), luteinizing hormone (LH) and E2 levels during LHRHa treatment for analytical limits of the test and cross-reactivity issues with tamoxifen administration. Nevertheless, particularly for premenopausal patients receiving LHRHa plus AIs, FSH, LH and E2 levels should be performed before the administration of AIs, to ensure effective OFS. A periodical monitoring through gynecological ultrasound evaluation was also suggested by some panellists.

C. *Potential predictive factors for suboptimal ovarian suppression are high body mass index (BMI), no prior chemotherapy, and low baseline FSH and LH levels*

According to the SOFT-EST trial results, the panellists considered low baseline FSH and LH levels, high BMI and no prior adjuvant chemotherapy as predictive factors potentially associated with suboptimal OFS [56].

D. *Surgical oophorectomy could be an alternative to LHRHa therapy, according to patients’ preferences.*

Surgical oophorectomy may be considered in women with severe side effects associated with OFS, no reproductive desire or approaching natural menopause

E. *LHRHa therapy should start preferably on the second day of the menstrual cycle*

Treatment with LHRHa should start in the early follicular phase (day 1–2 of the menstrual cycle) to increase the likelihood of obtaining OFS in a short time and reduce the risk of hyperstimulation or ovarian cysts formation [57].

F. *AIs should be administered at least 4 weeks after the first injection of LHRHa therapy, when associated with OFS*

AIs must be used only in combination with OFS in premenopausal women, since they cause negative hypothalamic feedback and ovarian stimulation. Therefore, their use should start only after an adequate period after the first dose of LHRHa to ensure effective OFS.

G. *Perimenopausal patients with chemotherapy-induced amenorrhea should receive OFS if baseline FSH and E2 levels are not in the postmenopausal range*

This statement was added after public discussion and submitted for final vote. This recommendation should warn clinicians that AIs are effective only in postmenopausal women or in a setting of ovarian ablation. Therefore, in case of chemotherapy-induced amenorrhea with FSH and E2 levels not clearly postmenopausal, exemestane must only be used in combination with OFS. Nevertheless, in case of ambiguity regarding the ovarian function (e.g. suboptimal ovarian suppression, incomplete compliance with LHRHa), tamoxifen in combination of OFS should be preferred, regardless of the ovarian function status.

## Conclusions

The ERA project represents an Italian perspective on the role of OFS for the adjuvant treatment of premenopausal patients with HR-positive EBC. Overall, in this study, 31 Italian oncologists reached a positive consensus over 28 statements related to prognostic groups, therapeutic strategy and ovarian function suppression in premenopausal patients with HR-positive EBC (Table 1). The highest level of positive agreement was reached with regard to the efficacy of OFS in addition to standard adjuvant endocrine therapy for premenopausal women with HR–positive EBC at intermediate or high risk of recurrence, with all the statements related to this topic exceeding the 97% of positive agreement. Of note, the evidence-based approach of the Steering Committee in defining the statements voted by the panellists may have limited contrasting positions among the expert panel, particularly when discussing objective results of the SOFT and TEXT trials. Indeed, consensus was reached, but with a lower level of agreement, on topics related to the definition of prognostic factors and their relevance in guiding therapeutic choices (> 84%) and ovarian function suppression in clinical practice (> 74%).

An update of the SOFT trial and the SOFT/TEXT joint analysis presented after the conclusion of this consensus study have recently confirmed the benefit of OFS in this setting with additional follow-up [7]. In conclusion, the SOFT and TEXT trials have changed the standard adjuvant treatment of premenopausal patients with HR-positive EBC, but the available treatment options require a careful risk assessment and toxicities evaluation to ensure the greatest clinical benefit for each patient.

## Abbreviations

AJCC: American Joint Commission on Cancer
ASCO: American Society of Clinical Oncology
BC: Breast cancer
BCFI: Breast cancer-free interval
BMI: Body mass index
CAP: College of American Pathologists
DDFS: Distant disease-free survival
DFS: Disease-free survival
E2: Beta-2-estradiol
EBC: Early breast cancer
ER: Estrogen-receptor
FSH: Follicle-stimulating hormone
HER2: Human epidermal growth factor receptor 2
HR: Hormone receptors
LH: Luteinizing hormone
LHRHa: Luteinizing hormone-releasing hormone analogs
OFS: Ovarian function suppression
OS: Overall survival
PgR: Progesterone-receptor
TNBC: Triple-negative breast cancer
TNM: Primary tumor, lymph node, and metastasis
